# Candida Contamination in Kidney and Liver Organ Preservation Solution: Does It Matter?

**DOI:** 10.3390/jcm10092022

**Published:** 2021-05-09

**Authors:** Sabrina Stern, Dmitri Bezinover, Peter-M. Rath, Andreas Paul, Fuat H. Saner

**Affiliations:** 1 Department of General, Visceral and Transplant Surgery, University Duisburg-Essen, 45147 Essen, Germany; Sabrina.stern@uk-essen.de (S.S.); andreas.paul@uk-essen.de (A.P.); 2 Anesthesiology and Perioperative Medicine, Penn State University, PA 17033, USA; bezinover@pennstatehealth.psu.edu; 3 Institute for Microbiology, University Duisburg-Essen, 45147 Essen, Germany; peter.rath@uk-essen.de

**Keywords:** *Candida*, preservation fluid, infection, liver, kidney, transplantation

## Abstract

Introduction: Fungal infections remain a major challenge affecting outcomes after kidney (KT) and liver transplantation (LT). Methods: In this retrospective single center study, the incidence of Candida contamination in renal and hepatic graft preservation solution (PS) was evaluated. In addition, Candida associated infections in recipients and related complications were analyzed. Results: Overall, the PS of 1248 hepatic and 1273 renal grafts were evaluated. The incidence of fungal contamination in the PS of hepatic and renal grafts was 1.2% and 0.86%, respectively. Additionally, the hepatic PS of one patient who underwent a combined liver–kidney transplant had Candida contamination. *Candida albicans* was the most common organism (70.4%) and 65.4% of the patients received antifungal treatment. *Candida*-associated complications in the recipients was 19%. Complications in LT patients included *Candida* peritonitis and *Candida* sepsis. Two KT recipients with contaminated PS developed a mycotic aneurysm at the anastomotic site resulting in severe bleeding. The 1-year mortality in patients with PS contamination for LT and KT recipients was 33% and 18%, respectively. Although the incidence of fungal contamination of PS was low, contaminated PS was associated with a high mortality. Conclusion: The results of the study suggest that PS should be evaluated for fungal growth.

## 1. Introduction

Transmission of donor derived infections is a significant problem and is associated with significant morbidity and mortality [1]. Invasive fungal infections are associated with poor outcomes [2], particularly if graft contamination progresses to a systemic infection in the recipient with the subsequent development of a mycotic aneurysm at a vascular anastomosis site that results in severe bleeding [3]. Echenique et al. [1] demonstrated the incidence of transmission for all kinds of infections (bacterial, viral, and fungal) from donors to recipients was as high as 0.2% [1]. Another study by Kovacs et al. reported a transmission rate for all organ transplants of between 0.2–1.7% [4]. These transmissions, however, usually resulted only in recipient contamination and did not progress to an infection affecting patient or graft survival.

It has been demonstrated that the source of most of these infections result from the use of contaminated preservative solution (PS) [5,6].

Overall, data regarding postoperative morbidity and mortality of recipients receiving a graft with fungal contamination are controversial. Some studies have demonstrated that PS contamination with *Candida species* can be associated with a high mortality [7]. Significant complications, as previously mentioned, include the development of mycotic aneurysms and rupture of anastomoses in both renal and hepatic grafts [8,9]. Other authors did not find an association between graft or PS contamination and recipient outcome [10].

Even though the incidence of fungal contamination in PS seems to be low, the association with significant life-threatening complications raises questions whether there should be routine PS evaluation and or prophylactic treatment of recipients receiving a graft found to be preserved in contaminated PS.

The aim of our retrospective study was to evaluate the incidence of fungal contamination of PSs and determine if there is an association with the use of contaminated PSs and infectious complications in KT and LT recipients.

## 2. Patients and Methods

This retrospective, single-center, cohort study was approved by the local ethics committee and followed the Declaration of Helsinki. The ethics committee waived informed consent due to the retrospective design of this study. Organ procurement was standardized according to guidelines of the German Foundation of Organ Transplantation (DSO = Deutsche Stiftung für Organtransplantation). Recently, Heise et al. [11] summarized the actual German guidelines.

All liver transplantations were performed using standard surgical techniques (cava replaced technique, non-piggy-back, first retrograde perfused via vena cava, no veno-venous bypass) and a standardized anesthesia protocol was applied to all patients. Patients were treated postoperatively in a single intensive care unit, applying standardized care consisting of triple immune suppression (corticosteroids, mycophenolate mofetile (MMF), and tacrolimus (TAC)). Daily trough levels of the calcineurininhibitors (CNI) (TAC) were measured for adjusting the daily dose (targeted TAC level 5–8 µg/L). Postoperatively antibiotic and antifungal prophylaxis was not applied, since there were no signs of infection, regardless if the patient was a high-risk patient for fungal infection or not. As perioperative antibiotic prophylaxis in liver transplant patients, we used single-shot ampicillin/sulbactam (3 g). If the transplant operation lasted longer than 3 h, a second dose was applied. We did not prolong the prophylaxis for 2 or 3 days. This approach was confirmed by an RCT conducted by Berry et al. [12]. This study clearly demonstrates no benefit for extension of the antibiotic treatment.

Kidney transplantation was performed by extraperitoneal access. Left kidney was transplanted in the left fossa obturatoria and vice versa. Vascular anastomosis was applied to the common iliac artery and vein. Ureteral anastomosis was performed in the Lich–Gregoir technique with placement of an internal (double J) stent. Most of the patients went to a peripheral ward, not to the ICU. Immunosuppression was induced by Basiliximab (Simulect™), a soluble Il 2 receptor antagonist and a triple immune suppression (corticosteroids, mycophenolate mofetile (MMF), and tacrolimus (TAC)). Daily trough levels of the calcineurininhibitors (CNI) (TAC) were measured for adjusting the daily dose (targeted TAC level 5–8 µg/L). Like the liver transplant patient, the kidney transplant patient did not receive a prolonged antibiotic or fungal prophylaxis. As the prophylaxis kidney recipient received only a single shot antibiotic with Cefazolin 2 g.

Beside that, we did not make any antifungal prophylaxis, we did not follow a specific protocol for fungal contaminated PS.

Between 1/2008 and 1/2019, the PS of 2521 KT and LT patients was evaluated.

Samples of PS (10 mL) were obtained during back table graft preparation, immediately after opening the organ bag. Samples were cultured for both aerobic and anaerobic strains over a period of 14 days. *Candida albicans* was identified within 48 h. Microbial charts were reviewed for PS contamination with *Candida species.* Recorded donor data included age, reason for donor brain death determination (donation after cardiac death donors are not used in Germany), vasopressor support, cold- and warm-ischemia time (CIT) and (WIT), and laboratory studies of liver function, coagulation, and serum creatinine.

Recipient information for both, liver and kidney transplanted patients, was collected from their medical records and included age, gender, cause of end stage liver disease, laboratory values and for LT patients, calculated MELD score.

Postoperative complications were assessed by the Dindo–Calvian score [13]. Antifungal treatment was also evaluated. In general, routine antifungal prophylaxis was not used in any recipient. This was independent of the MELD score or intraoperative course. Only if patients developed signs or was highly suspected to have a fungal infection, was antifungal treatment started. Patients with signs of Candida sepsis were treated with either caspofungin or echinocandin.

## 3. Results

For the purpose of this study, the incidence of graft contamination and recipient associated complications were analyzed separately for LT and KT.

### 3.1. Liver Recipients

Fifteen recipients received grafts preserved in PS contaminated with Candida species (15/1248, 1.2%). *Candida albicans* was found in 10 cases, and Candida *tropicalis* in 5 cases. Eight cases of additional bacterial contamination were recorded (Table 1). Among the 15 patients receiving PS contaminated with Candida, 20% developed direct Candida-related complications (2 with Candida peritonitis and 1 with Candida sepsis). Six patients (40%), including one with a proven *Candida* infection, developed dialysis-dependent kidney failure. Seven patients (47%) required re-operation due to bleeding (4 cases), surgical site infection (2 cases), and fascial dehiscence (1 case). Among the four patients requiring re-operation for bleeding, three had a confirmed *Candida* infection. None of the re-operated patients experienced a mycotic aneurysm or mycotic-related thrombosis.

Four of 15 patients receiving a graft preserved in a solution contaminated with *Candida* died during their hospital stay. These cases included 2 patients that developed severe sepsis and septic shock with multiorgan failure, 1 patient with cirrhotic cardiomyopathy and subsequent cardiogenic shock, and one intraoperative death after 2 h of cardiopulmonary resuscitation. A fungal infection was not confirmed in any of these patients. Early allograft dysfunction, according to the definition by Olthoff et al. [14], occurred in 8 cases (53%), and there were no grafts with primary non-function.

Antimycotic treatment was started in 80% of all patients receiving a graft preserved in a contaminated PS (due to suspicion of infection), and in 100% of those with a confirmed infection. The median start of treatment was on the postoperative day (POD) 3 (0–12), with a median duration of 9 days (2–24). The follow-ups in liver recipients were minimum 1 day, maximum 3579 days, and median 1054 days.

According the Dindo–Clavien classification, Grade III and above complications occurred in 11 patients (73%). Rejection was recorded in 2 cases (13%).

The median ICU stay was 8 days (1–31 days), while median time on a ventilator was 44 h (8–359). The longest ventilation time, 359 h, was recorded for a patient with Candida sepsis. The median hospital stay was 25 days (7–79 days). All patients with a proven Candida infection survived.

At the end of follow-up (19 September 2019), 53% of patients transplanted with a graft preserved in contaminated PS survived. In the patients who died postoperatively, the deaths occurred within the first week after transplantation and all had a functioning graft. The overall one-year survival was 67% (10/15).

### 3.2. Donors for Liver Transplantation with Contaminated PS

Donor data are presented in Table 2. The main diagnosis leading to brain death was cerebro-vascular accident (53% of cases). Calculated median donor risk index was 1.8 (1.2–2.3). Ten donors received antimicrobial, but not antimycotic, therapy. Median ICU stay was 3 days (1–19). No donor was suspected of having an ongoing infection. Median white blood count (WBC) was 14.5 × 10^9^/L (4.5–20.7 × 10^9^/L) and c-reactive protein (CRP) was 5.5 mg/dl (1.7–29.5 mg/dL) immediately before organ recovery. Noradrenaline (NA) was used for vasopressor support in all donors: 9/15 received a dose <0.1 µg/kg/min, 4/15 required NA dose between 0.1–0.5 µg/kg/min, and in 2/15 NA dose was higher than 0.5 µg/kg/min.

Thirteen of 15 donors (80%) met extended donor criteria (ECD).

The most used PS was histidine-tryptophan-ketoglutarate (HTK) followed by University of Wisconsin (UW) solution. Median CIT was 8 h (6 h 29 min–10 h 57 min) and WIT time, 32 min (22–56 min).

### 3.3. Kidney Recipients

Eleven recipients received grafts preserved in PS contaminated with Candida species (11/1273, 0.86%). Detailed information is provided in Table 3. For patients undergoing dialysis before transplantation, the median duration was 40 months (0–184). Delayed graft function occurred in 27% (3/11) of cases. Rejection was recorded in 2 cases. Antimycotic treatment was started in 5 of 11 cases due to suspicion of infection.

There were several complications among recipients receiving organs preserved in contaminated PS. Two of eleven patients developed a Candida-associated infection at the arterial anastomosis. The first patient underwent an en-bloc transplantation of 2 pediatric kidneys. On POD 9, the patient suddenly developed hemorrhagic shock and died after 2 h of cardio-pulmonary resuscitation. Post-mortem examination demonstrated an arterial anastomosis leak and significant retroperitoneal hematoma. Candida infection at the site of the vascular anastomosis was confirmed.

In the second patient, radiological imaging, prompted by symptoms of ureteral compression and urinary retention, demonstrated a partial thrombotic aneurysm at the arterial anastomosis. The patient underwent a nephrectomy where intraoperative microbial testing demonstrated the same *Candida species* seen in the culture of the PS used for the patients graft. The patient died 225 days after transplantation due to recurrent sepsis.

One other patient, who also received an en-bloc transplantation of 2 pediatric kidneys, underwent removal of the upper kidney graft 9 months after transplantation due to a suspicion of infection. Histological work-up, however, demonstrated a lymphoma and no Candida infection.

In these patients, the Dindo–Clavian score was ≥3 in 45% (5/11) of patients, whereas in patients without Candida related complications, it was 0%. The median serum creatinine 6 months after transplantation in surviving patients was 1.5 mg/dl. The follow-ups in kidney recipients were minimum 9 day, maximum 3763 days, and median 1005 days.

### 3.4. Donors for Kidney Transplantation with Contaminated PS

There were 11 donor grafts preserved in contaminated PS. Donor data are presented in Table 4. Seven out of eleven kidney donor were multi-organ-procurements. The main causes for patient death were cerebral vascular accident (CVA) and anoxia (45% each). One other patient became an organ donor after severe multi-organ trauma but with preserved kidneys. ECD criteria for kidneys were fulfilled in 27% (3/11) of cases. Two donors were diagnosed with pneumonia, and another donor experienced streptococcus-related sepsis. Antibiotic treatment was used in 27% (3/11) of all donors.

Two donors underwent surgery for gastric ulcer perforation 7 days prior to the diagnosis of brain death. Gastric injury was identified incidentally in one case during organ recovery. Median ICU stay was 7 days (2–12).

Median c-reactive protein (CRP) levels immediately before harvesting was 11.1 mg/dl (0.3–27.6) and white blood cells (WBC) was 13.6 × 10^9^ (6.2–30.1 × 10^9^). Vasopressor support in 8 donors was <0.1 µg/kg/min and in 3 donors between 0.1–0.5 µg/kg/min.

The most frequently used PS was HTK in 73% (9/11) cases (including combined LKT), celsior solution (CS) in one case, and UW in one donor. Median CIT was 12 h 30 min (8 h 24 min-28 h) and median WIT, 29 min (18–60).

### 3.5. Combined Liver–Kidney Transplant Donor and Recipient

One patient underwent combined liver–kidney transplant due to Joubert syndrome. The 23-year-old recipient had been on dialysis for 28 months prior to transplant. Only the PS of the liver graft was contaminated with *Candida albicans.* HTK PS was used in this case.

The kidney graft, obtained from a different patient, was preserved in PS found to be contaminated with *Staphylococcus epidermidis* and *Enterococcus faecium*. For the liver, the CIT was 6 h and 30 min and WIT 24 min. For the kidney, the CIT was 9 h and WIT 16 min, respectively. Postoperative immunosuppression included tacrolimus, prednisone, and mycophenolate mofetil. Both organs had excellent post-transplant function. The patient did not receive antifungal treatment at any time.

## 4. Discussion

We evaluated a total of 2521 PSs and found that 1.2% (15/1248) of liver grafts and 0.86% (11/1273) of renal grafts were found to have been preserved with a PS that had fungal contamination. One other patient received a combined liver and kidney transplantation where only the PS of the liver graft was contaminated. *Candida species* were the most frequently found yeast in the PSs evaluated.

PS fungal contamination was associated with a high incidence of serious complications. In patients with liver grafts preserved in contaminated PS, 20% developed Candida-related complications and required re-operation. All of these patients, however, survived and were discharged from the hospital.

In the kidney group, 18% of patients experienced Candida-related complications. Both patients developed life-threatening bleeding related to a mycotic aneurysm at the arterial anastomosis. One patient died during their hospitalization and the other 7.5 months later due to Candida sepsis.

Fungal contamination of preservation solution is a well-known problem. Botterel et al. [15] evaluated the PS used in 728 transplants and found that the yeast contamination rate was 0% (0/62), 3.1% (11/356), and 4.1% (10/241) for heart, kidney, and liver grafts, respectively [15]. These findings were confirmed by Singh et al. who reported an incidence of 3.7% for kidney grafts and 4% for liver grafts [16].

The progression from contaminated PS to contaminated graft to significant infection is frequently associated with worse outcomes and is well recognized and problematic for both LT and KT. Janny et al. performed a retrospective analysis of PSs with respect to bacterial and fungal contamination in 477 liver grafts from deceased donors [5]. Forty-five (9.5%) PSs were found to be contaminated with 1 or 2 pathogens. Systemic infections associated with the specific pathogen cultured from the PS occurred in 8 (18%) of 45 recipients.

There are several reports of mycotic aneurysms forming after transplantation in the hepatic artery of liver grafts preserved in *Candida albicans* contaminated PS [9,17]. Levesque et al. reported a case of severe hemorrhagic shock due to rupture of the hepatic artery anastomosis shortly after LT [9]. A swab of the hepatic artery was positive for *Candida albicans.* The same strain was found in the PS of the graft. A retrospective multicenter study, involving 6 French centers, evaluated all cases of *Candida* species infections related to the use of contaminated PS [18]. Similar to our study, the authors reported an incidence of *Candida* species contamination of 1.2%. The most common yeast found was *Candida albicans.* In this study, approximately 80% of recipients received antifungal treatment of any kind, but primarily with echinocandins. Among the entire group, 28.6% developed yeast-related complications including hepatic artery aneurysm. One-year mortality in patients with fungal complications was 62.5%, primarily related to bleeding.

PS yeast contamination in kidney grafts is also well described with an incidence between 0.4 and 8.6% [5,15,19], resulting in significant complications than can be life-threatening [8,10]. In a single-center prospective cohort of 70 patients undergoing KT, Rodrigues et al. found that 6 (8.6%) PS samples were positive for yeast (4 *Candida albicans* and 2 *Candida glabrata)*. Two recipients developed severe mycotic vascular complications leading to transplant nephrectomy [19]. Mai et al. [8] reported 4 cases of a local mycotic aneurysm forming at the vascular anastomotic site in renal grafts preserved with Candida contaminated PS [8]. In 3 of these cases, the aneurysms ruptured, causing severe bleeding and resulting in 2 deaths. The fourth patient underwent a preemptive nephrectomy during which the aneurysm ruptured. In our patient cohort, bleeding occurred within the first 4 weeks. This is in line with the report of Mai et al. [8]. Most kidney transplanted patients are discharged from the hospital within 10 days. Recipients with *candida spp.* swab from the PS require a careful follow-up.

Taking into consideration the severity of fungal-associated complications, identification of factors predisposing to the development of yeast infections is critical. A recent study from our group identified that a WBC ≥ 9.1 × 10^9^/L in the donor was an independent risk factor for contamination [6]. Although there were no overt signs of infection in our current cohort, the median WBC and CRP levels were increased.

Fungal contamination of PS usually occurs during graft harvesting. Trauma patients are a known high-risk group for fungal contamination, particularly in donors with gastrointestinal tract injury [16,20]. In our study, the esophagus was perforated during harvesting in one LT donor, while in the kidney group, two donors underwent gastric ulcer repair 7 days prior to organ recovery. In one other donor, gastric perforation during harvesting was noted in the operative report. PS contamination can also occur as a result of damage to the organ bag or during back-table preparation [4,5,6,19,21,22].

The use of antibiotics can also affect the development of fungal infections. The study of Levesque et al. demonstrated that the use of antibiotics in donors is a risk factor for yeast contamination of the graft [18]. The authors postulated that the antibiotics administered may have allowed yeast overgrowth in the donors’ gastrointestinal tract. The highest incidence was with the use of carbapenems [23]. The development of multi-resistant bacteria in the gastro-intestinal tract after one week of imipenem use has been reported to be as high as 5.6% [24].

In our cohort, the use of antibiotics was reported in 66.6% among liver donors, and in 72.7% in the kidney group, which are lower than previously described. This is potentially one of the reasons why patients in our study had a lower incidence of fungal contamination compared to published reports. However, this explanation is speculative and needs further evaluation.

Although not routinely performed, evaluation of PS for contamination seems prudent. Riuz et al. [21] found positive cultures in samples of PS obtained in 98% of patients, although most of them (75%) were superficial saprophytic flora such as coagulase *negative staphylococci*, *Streptococcus viridans*, and C*orynebacterium species* [21]. In this study, microorganisms isolated from post-transplant cultures did not match the bacterial or fungal flora obtained from the PS used with the graft. Nevertheless, considering the consequences of infection in immunocompromised recipients, the authors concluded that routine culturing of PS should be done. Oriol et al. performed a prospective trial culturing donor graft PS, blood, and bile, as well as recipient ascitic fluid [25]. The incidence of contaminated PS was 92% (46 of 50 cases), but only 28% (14/50) were contaminated by recognized pathogens. Blood and bile cultures from the donor were positive in 28% and 6% of cases, respectively, whereas ascitic fluid was positive in 22% of cases. No LT recipient developed an infection due to the transmission of the infectious organism isolated from the PS. In a subsequent meta-analysis, the same group confirmed a high incidence of PS contamination with a rate of infection transmission of 1.3% [22]. These results confirmed the importance of routine PS microbiological evaluation, considering the potential for life-threatening complications.

Another important question to answer is, if patients received a yeast contaminated graft from any source, do they require routine antimycotic treatment. In a retrospective analysis, Janny et al. [5] found that infections occurred significantly less frequently in recipients receiving appropriate antimicrobial therapy than in those without (41% vs. 3.8%, *p* = 0.005). The authors concluded that swabs from every PS should be performed and if contamination was found, treatment with antibiotics should be started [5]. There are guidelines available describing antifungal prophylaxis and treatment of high-risk patients (defined as patients with a MELD ≥ 30, or requiring reoperation due to bleeding or bile leak, postoperative renal-replacement treatment or receiving high-dose cortisone due to rejection) [26,27]. However, no guidelines or consensus are available for antimicrobial management solely related to PS contamination, although many experts favor antifungal treatment [16]. It appears that antifungal treatment could be recommended in high-risk patients [27] and if donors have laboratory signs or symptoms of infection [6]. The detection of (1–3)-ß-D-glucan in serum might be useful to identify patients with invasive infection. A meta-analysis (mainly patients with hematological malignancies and solid organ tumors) showed a sensitivity of 80% and a specificity of 63% [28]. A study with liver transplant patients showed a sensitivity of 83% and a specificity of 89% when combining results of a (1–3)-ß-D-glucan assay and a colonization index of ≥0.5 [29]. In lung transplant patients, the specificity was quite lower (9%) [30]. Our experience with serial tests after liver transplantation indicate that (1–3)-ß-D-glucan remains detectable in many patients for 2–4 weeks after transplantation and is therefore not useful to identify patients with early Candida infection (unpublished data).

In the absence of documented infection, empiric treatment should last 2 weeks. In patients who develop clinical or microbiological evidence of infection, treatment should be extended to 4–6 weeks, and for patients with vascular involvement, treatment for at least 6 weeks has been recommended [16].

In our study cohort, the most frequently demonstrated yeast was *Candida albicans*. This was followed by *Candida krusei* or *glabrata.* These organisms are usually treated primarily with echinocandins. After performing susceptibility tests, de-escalation to fluconazole in uncomplicated cases could be considered. We also recommend prophylactic antifungal treatment for more than 2 weeks if PS contamination was confirmed. Treatment can be discontinued if the patient is clear from any signs of infection.

It remains unclear whether a recipient receiving a graft preserved with yeast contaminated PS, but with functioning organs and an unremarkable clinical course, should undergo radiologic follow-up to exclude the development of a mycotic aneurysm. In our evaluation of 27 patients, only 2 KT recipients experienced vascular complications. Taking into consideration that these complications were devastating, we recommend the establishment of radiologic follow-up protocols.

This study has several limitations. It is a retrospective analysis and can demonstrate only potential associations, not causality. The reason for this is that the cause of PS contamination could not be clearly determined. Liver and kidney transplant patients were managed by different services and with different methods of prophylaxis, and treatment was not standardized. The strength of this study is that all components of the organ recovery and transplant procedures were standardized and followed a strict institutional protocol. The high number of PSs from one center, analyzed in the same laboratory, also support validity of our results.

In conclusion, yeast contamination of PS can lead to life-threatening complications. Routine PS culturing should be performed. In addition, routine recipient antifungal therapy and aggressive postoperative screening for mycotic aneurysms can potentially prevent life-threatening complications.

## Figures and Tables

**Table 1:** Liver recipient data.

Recipient	Age/Gender	Underlying Disease	Meld	Content Preservation Fluid	Antimycotic Treatment	Start and Duration Treatment (POD/Days)	Candida-Associated Complication	Survived (Follow-Up in Days)
1	55/m	Alcohol	10	C. Tropicalis	-	-	-	1241
2	56/m	Hepatitis C	17	C. albicans Lactobacillus sp. Bifidobacterium sp.	Fluconazol	6/14	-	1865
3	54/f	Cryptogenic/Alcohol	17	C. albicans	-	-	-	3579
4	10/m	Bile duct atresia	10	C. albicans Citrobacter brakii Citribacter amalonaticus Klebsiella pn.	Fluconazol	4/24	Candida-peritonitis	2912
5	33/f	Autoimmunhepatitis	25	C. albicans Lactobacillus acidophilus	First line Caspofungin/then Fluconazol	5/10	-	2223
6	55/f	Acute liver failure	31	C. tropicalis Proteus mirabilis	Fluconazol	3/5	-	7
7	32/f	Acute liver failure	37	C. albicans	Caspofungin Fluconazol	2/11	Candida-sepsis	1992
8	62/f	Primary bilary cirrhosis	10	C. lusitaniae	Caspofungin	3/4	-	6
9	27/f	Wilson disease	30	C. krusei	Caspofungin	3/10	-	J(1692)
10	53/m	Alcohol	10	C. albicans Streptococcus salivarius Streptococcus mitis Streptococcus oralis	Fluconazol	2/4	-	628
11	37/m	Secondary sclerosing cholangitis	35	C. albicans Enterococcis faecium	Caspofungin Liposomal Amphotericin B	2/7	-	8
12	35/m	Alcohol	11	C. albicans	-	-	-	1559
13	38/m	Alcohol	22	C.albicans Staphylococcus epidermidis	Caspofungin	5/4	-	1013
14	0/m	Newborn Hemochromatosis	26	C. albicans	Fluconazol	0/2		1
15	44/m	Alcohol	22	C.glabrata Lactobacillus spp.	Caspofungin	12/15	Candida-peritonitis	255

MELD: model of end-stage-liver disease, PF: preservation fluid, C. = Candida, POD: Postoperative Day, m = male, f = female.

**Table 2:** Donor data of liver recipients.

Donor	Age/Gender	Cause of Death	DRI	ICU stay in Days	ECD	Use of Antibiotics	Kind of PF	WBC	Noradrenalin (µg/kg KG/min)	Dobutamin (µg/kg KG/min)	Dopamin (µg/kg KG/min)	Gastro-Intestinal Perforation
1	49/F	CVA	1.5	19	Yes	Yes	HTK	14.50	0.05			No
2	58/F	Trauma	1.3	3	Yes	No	HTK	10.00	0.07			Thoracic Esophagus Perfusion
3	59/F	Other	2.0	7	Yes	Yes	UW	12.00	-	2		No
4	7/M	Anoxia	1.2	2	Yes	No	HTK	4.50	-		9.78	No
5	50/M	CVA	2.0	7	Yes	Yes	HTK	15.20	0.05			No
6	65/F	CVA	2.3	2	Yes	No	UW	6.69	0.66		5	No
7	50/F	CVA	1.8	2	No	Yes	UW	20.70	0.20			No
8	1/F	Other	1.6	2	Yes	No	UW	11.00	0.08			No
9	56/F	CVA	1.9	3	No	No	UW	18.89	0.10			No
10	74/M	CVA	2.2	1	Yes	Yes	UW	9.40	0.20			No
11	38/M	Anoxia	1.4	5	Yes	Yes	HTK	14.60	0.05			No
12	31/M	Anoxia	1.2	10	Yes	Yes	HTK	18.10	0.02			No
13	45/W	Anoxia	1.6	8	Yes	Yes	HTK	12.40	0.53			No
14	30/F	CVA	2.3	2	No	Yes	HTK	19.70	0.15			No
15	69/M	CVA	2.0	2	Yes	Yes	IGL-1	20.40	0.20			No

DRI: Donor risk index; ICU: intensive care unit; ECD: extended criteria donor; PF: preservation fluid. CVA: cerebro-vascular accident; M: male; F: female; HTK: histidine-tryptophan-ketoglutarate; UW: University of Wisconsin, IGL 1: Institut Georges Lopez-1.

**Table 3:** Kidney recipients, underlying disease, results culture of preservation fluid.

Recipient	Age/Gender	Underlying Disease	Culture Result Preservation Fluid	Antimycotic Treatment	Candida-Associated Complication	Survival (Follow-up in Days)
1	71/M	Alport Syndrome	C. albicans	Fluconazole	-	3605
2	5/F	Congenital kidney dysplasia	C. parapsilosis Staphyloccous epidermidis	-	-	3763
3	52/F	Diabetic nephropathy	C. albicans	Fluconazole	-	1916
4	55/M	Henoch Schönlein purpura (HSP)	C. albicans	-	-	3390
5	44/M	Polycystic kidney disease	C. albicans C. tropicalis	-	Bleeding arterial anastomsis	9
6	46/F	Diabetic nephropathy	C. albicans	-	-	1208
7	60/F	Chronic glomerolonephritis	C. parapsilosis	-	-	1156
8	62/M	Diabetic nephropathy	C. albicans Propionibacterium acnes	Caspofungin Fluconazole	Aneurysm anastomosis Candida sepsis	225
9	49/M	Chronic glomerolonephritis	C. albicans	-	-	853
10	54/F	Shrink kidney disease	C. albicans	Fluconazole	-	618
11	22/M	Cystinosis (lysosomal storage disease)	C. albicans Stenotrophomonas maltophilia Staphyloccous epidermidis	Fluconazole	-	565

**Table 4:** Associated donor data of kidney recipients.

Donor	Age/Gender	Cause of Death	ICU Stay	Multi-Organ Procurements Yes/No	ECD	Use of Antibiotics	Kind of Preservation Fluid	WBC	Noradrenalin (µg/kg KG/min)	Dopamin (µg/kg KG/min)	Perforation
1	68/F	Anoxia	4	No	Yes	Yes	HTK	13.60	0.025		No
2	10/M	CVA	7	Yes	No	No	HTK	18.20	0.27		No
3	58/F	CVA	2	No	Yes	No	CS	6.24	–	3	No
4	53/F	CVA	12	No	No	Yes	HTK	30.10	0.25		Ulcus duodeni perforation 7 days ahead brain dead
5	0/M	Trauma	2	Yes	No	Yes	HTK	10.00	0.08		No
6	42/F	Anoxia	6	Yes	No	Yes	HTK	19.40	0.01		Ulcus duodeni perforation 7 days ahead brain dead
7	0/F	Anoxia	2	Yes	No	Yes	Unknown	10.78	–		No
8	31/M	Anoxia	10	Yes	No	Yes	HTK	18.10	0.02		No
9	43/F	CVA	7	Yes	No	Yes	HTK	16.10	0.33		No
10	2/M	Anoxia	9	Yes	No	No	HTK	7.00	–		No
11	59/M	CVA	6	No	Yes	Yes	UW	8.60	0.02		Gastric injured during harvesting

F = female, M = male, ICU: intensive care unit, ECD: extended criteria donor, HTK: histidine-tryptophane- ketoglutarate, UW: University Wisconsin, CS = Celsior.

## Data Availability

The datasets generated and/or analyzed during the current study are not publicly available due to privacy patients’.

## Abbreviations

**CIT**	Cold ischemia time
**CRP**	C-reactive protein
**CVA**	Cerebral vascular event
**DSO**	Deutsche Stiftung für Organtransplantation
**ECD**	Extended criteria donor
**HTK**	Histidine-tryptophan-ketoglutarate
**KT**	Kidney transplantation
**LT**	Liver transplantation
**MELD**	Model of end-stage-liver-disease
**PS**	preservation fluid
**UW**	University of Wisconsin
**WIT**	Warm ischemia time
**WBC**	White blood count
